# Variation in wing pattern and palatability in a female-limited polymorphic mimicry system

**DOI:** 10.1002/ece3.1308

**Published:** 2014-11-20

**Authors:** Elizabeth C Long, Thomas P Hahn, Arthur M Shapiro

**Affiliations:** 1 Center for Population Biology, University of California DavisDavis, California; 2 Entomology Section, Natural History Museum of Los Angeles CountyLos Angeles, California; 3 UCLA La Kretz Center for California Conservation Science, Institute of the Environment and Sustainability, University of CaliforniaLos Angeles, California; 4 Department of Neurobiology, Physiology, and Behavior, University of California DavisDavis, California; 5 Department of Evolution and Ecology, University of California DavisDavis, California

**Keywords:** Batesian mimicry, *Chlosyne hoffmanni*, *Chlosyne palla*, *Euphydryas chalcedona*, palatability

## Abstract

Checkerspot butterflies in the genera *Euphydryas* and *Chlosyne* exhibit phenotypic polymorphisms along a well-defined latitudinal and elevational gradient in California. The patterns of phenotypic variation in *Euphydryas chalcedona, Chlosyne palla,* and *Chlosyne hoffmanni* suggest a mimetic relationship; in addition, the specific patterns of variation in *C. palla* suggest a female-limited polymorphic mimicry system (FPM). However, the existence of polymorphic models runs counter to predictions of mimicry theory. Palatability trials were undertaken to assess whether or not the different color morphs of each species were distasteful or toxic to a generalized avian predator, the European starling (*Sturnus vulgaris*). Results indicate that the black morph of *E. chalcedona* is distasteful, but not toxic, to predators, while the red morph is palatable. C *. hoffmanni* and both color morphs of *C. palla* are palatable to predators. Predators that learn to reject black *E. chalcedona* also reject black *C. palla*, suggesting that the latter is a FPM of the former. *C. hoffmanni* does not appear to be involved in this mimetic relationship.

## Introduction

Mimicry theory predicts that once a predator guild begins to associate a warning signal (aposematism) with a penalty (noxiousness) of the model, individuals that exhibit the familiar aposematic phenotype will gain protection from predation and will experience increased fitness. Theory also predicts that selection will act to reduce variation in the aposematic phenotype of models as individuals that differ from that phenotype should suffer reduced fitness due to increased predation (Benson 1972; Harvey et al. 1982; Endler and Greenwood 1988). An opposing hypothesis proposes that models may evolve variability and thus “escape” from the mimic, termed coevolutionary chase (Huheey 1988; Joron and Mallet 1998). Because a fundamental tenet of Batesian mimicry holds that the mimic is detrimental to the model, particularly as the mimic increases in abundance, a model that is able to evolve and establish a new aposematic phenotype, free of mimics, would gain a selective advantage (Turner et al. 1984; Huheey 1988; Malcolm 1990; Joron and Mallet 1998). Although possible, a predicted increased rate of evolution in the mimic versus the model coupled with strong stabilizing selection on the model makes this an unlikely scenario (Joron and Mallet 1998).

In mimics, however, phenotypic polymorphism may in fact offer a selective advantage. In the case of female-limited polymorphic mimicry (FPM), males of the mimic species are nonmimetic while females are polymorphic, with some individuals exhibiting the mimetic, dishonest aposematic phenotype while others are nonmimetic. Proposed adaptive explanations for this subtype of Batesian mimicry include individual-level natural selection, kin selection, group selection, and sexual selection (Sheppard 1962; Burns 1966; Wickler 1968; Barrett 1976; Turner 1978; Wiklund and Jarvi 1982; Stamps and Gon 1983; Kunte 2009; Allen et al. 2011).

Numerous cases of mimicry have been described in butterflies, as have several examples of FPM systems (Bates 1862; Muller 1879; Ford 1936; Van Zandt Brower 1958a,b,c; Clarke and Sheppard 1962; Benson 1972; Bowers 1983a; Prudic et al. 2002). The most famous case of FPM mimicry occurs in *Papilio dardanus* Brown, where the females exhibit at least 14 different phenotypes, mimicking numerous models (Trimen 1868; Ford 1936; Nijhout 2003). However, in most of these described cases, the model shows little phenotypic variation, consistent with the theory. A notable exception occurs in the western United States, where *Euphydryas* checkerspots, which have been demonstrated to be unpalatable to generalized avian predators, show considerable phenotypic variation (Bowers 1981, 1983a,b; Bowers et al. 1985). In particular, the variable checkerspot, *Euphydryas chalcedona* Doubleday, true to its name, varies between populations so much that a casual observer may have difficulty recognizing individuals from populations 1000 m apart in elevation as conspecifics.

A putative FPM relationship exists between *E. chalcedona* (as the model) and the northern checkerspot, *Chlosyne palla* Boisduval (as the mimic). Like *E. chalcedona*, populations of *C. palla* show a great deal of wing pattern variation along a latitudinal and elevational gradient, but unlike the putative model, only females of the mimic vary. The phenotypic distribution of both species is complex, and there appears to be a strong correlation between the two (Shapiro and Manolis 2007). Other related sympatric species, *for example Chlosyne hoffmanni* Behr, strongly resemble some forms of both *E. chalcedona* and *C. palla* and could be involved in a complex mimicry ring. These relationships have not been previously tested experimentally and would represent an interesting advancement of our knowledge of both the FPM phenomenon as well as coevolutionary chase promoting polymorphism in the model. If confirmed, it would seem to be an elegant adaptive explanation for phenotypic variability of the putative model in this system, capturing an evolutionary snapshot of a cyclical coevolution (Joron and Mallet 1998).

In order to address the questions pertaining to phenotypic variability in both the model and the mimic in this putative mimicry system, we used a generalized avian predator to first test the palatability of three species of western checkerspot butterflies: two predominant phenotypic forms of *E. chalcedona*; the mimetic and malelike forms of *C. palla*; and *C. hoffmanni*. We addressed not only whether or not the butterflies were distasteful to predators but also whether they were in any way toxic. We then evaluated putative mimetic relationships between the species and discussed the possible explanations for the observed polymorphisms.

## Methods

### Butterfly species

**Figure 1:**
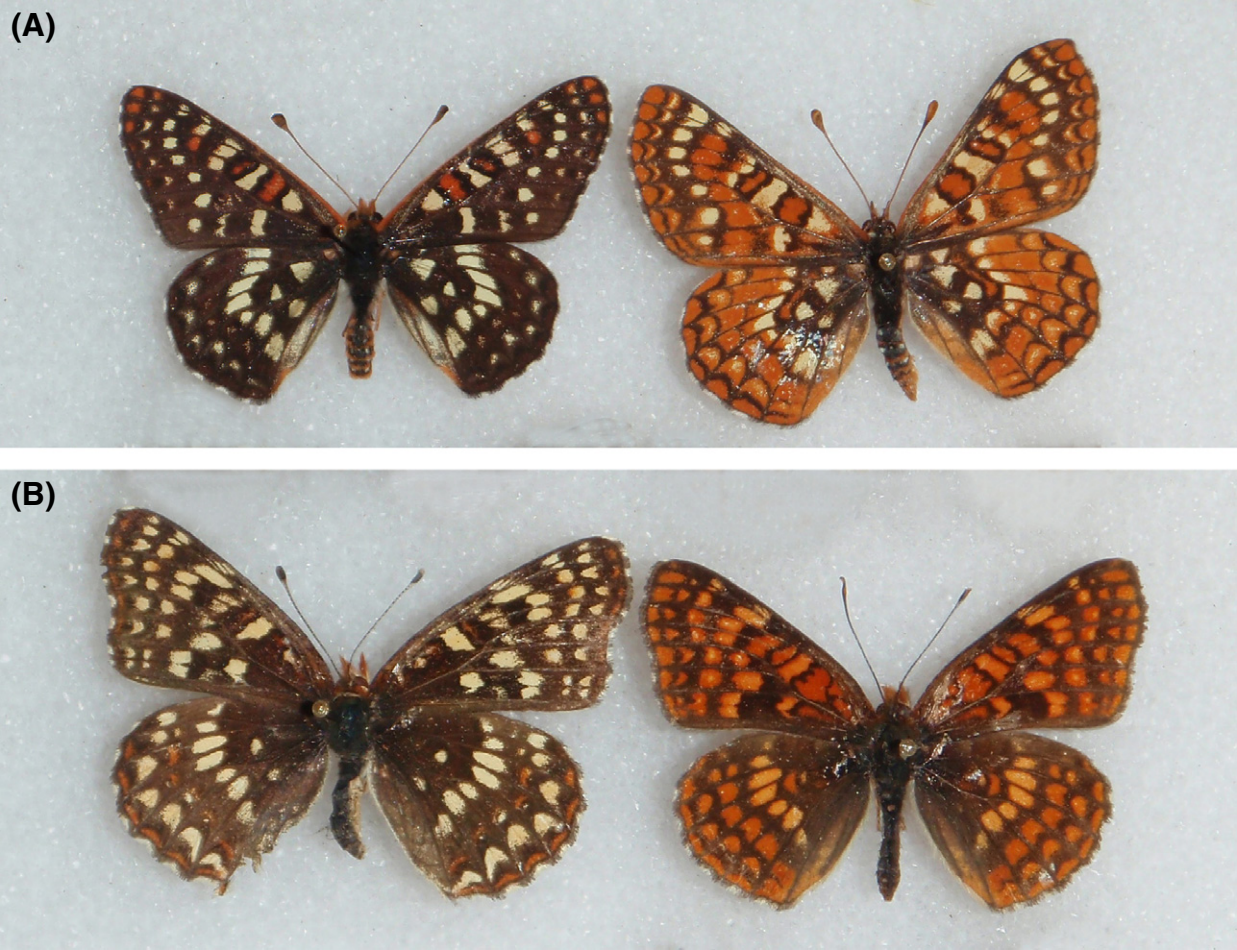
(A) (top row) Two predominant forms of *Euphydryas chalcedona*, black form (left), and red form (right). (B) (bottom row) Two predominant forms of *Chlosyne palla*, black form (left), and red form (right).

The putative model in the study system is *Euphydryas chalcedona*, the variable checkerspot. This species is widespread in the western United States, frequently found in rocky canyons, and is univoltine with a late spring – early summer flight season. Some populations are unpalatable to a generalized avian predator (Bowers 1981), supporting the hypothesis that this species acts as a model. The larvae sequester iridoid glycosides from host plants in at least part of its range although it does exhibit geographic variation in host plant usage; those chemical defenses are retained in adults (Bowers 1981, 1983a,b, 1986; Bowers and Puttick 1986; Stermitz et al. 1994). It also displays a great deal of phenotypic variation in wing pattern and color throughout its range, with an especially pronounced elevational difference in populations in California's Sierra Nevada Range. Below ∼1700 meters, populations are predominantly black and white with some red accents, while at higher elevations, populations show a strong red-orange background color with black pattern elements (Fig.1A, Table1). Phylogenetic evidence indicates that these forms do not represent separate species (Long et al. *,* 2014).

**Table 1:** Distribution of phenotypes of the three species of checkerspot butterflies used in the study. High elevation refers to populations above ∼1700 m, while low elevation refers to populations below ∼1700 m

	*Euphydryas chalcedona*		*Chlosyne palla*		*Chlosyne hoffmanni*	
	Male	Female	Male	Female	Male	Female
High Elev.	Red	Red	Red	Red	Red	Red
Low Elev.	Black	Black	Red	Red, Black, Int.	NA	NA

The putative mimic of *E. chalcedona* in this study is *Chlosyne palla*, the northern checkerspot. Like the model, populations of this species are red-orange at high elevations in the Sierra, while at lower elevations (and in other areas where the model is red-orange) males retain the red-orange phenotype while females are red-orange, black (similar to *E. chalcedona*), or intermediate between the two (Fig.1B, Table1). This species is thus a putative FPM of *E. chalcedona*. It is difficult to accurately assess female phenotypic ratios in these polymorphic populations due to sampling bias, but the black form appears to be the prevailing female phenotype in most areas (E.C.L. pers. obs.). Larvae of *C. palla* eat *Eurybia radulina* (Asteraceae) and occasionally *Solidago* (Asteraceae), neither of which has been shown to produce defensive chemicals. Field trials from other populations indicate this species is unlikely to sequester or produce defensive compounds (Ley and Watt 1989).

The third butterfly species in this study is *Chlosyne hoffmanni*, Hoffman's checkerspot. A close relative of the putative FPM mimic, *C. hoffmanni*, also bears a strong phenotypic resemblance to the red-orange *C. palla* form. The two species are often parapatric and sometimes sympatric, sharing similar flight seasons and life history strategies. This species does not co-occur with the black form of *E. chalcedona* but is often found within the range of the red-orange *E. chalcedona* form (Table1). In the study region, the host plant of *C. hoffmanni* is *Eucephalus breweri* (Asteraceae), which has not been shown to produce defensive chemicals. We included *C. hoffmanni* in this study to test whether it might also be a component of the putative *E. chalcedona – C. palla* mimicry system, either as a mimic or model of the red-orange forms of those species. If so, it could lend credence to the coevolutionary chase hypothesis, as an additional Batesian mimic would put added pressure on the model to escape.

The cabbage white butterfly, *Pieris rapae* Linnaeus, was used as a palatable control (Wourms and Wasserman 1985). This introduced species is widespread and common throughout the U.S., including our study area. Individuals were collected from around the vicinity of Davis, CA, where the caterpillars fed on *Lepidium latifolium* (Brassicaceae).

Black *E. chalcedona* were collected from one site in Healdsburg, Napa Co., CA, where the larval host plant is known to be *Scrophularia californica* (Scrophulariaceae) (and possibly sometimes *Diplacus aurantiacus* (Phrymaceae)); red *E. chalcedona* from Huntington Lake, Fresno Co., CA, where the host plant is *Penstemon newberryi* (Plantaginaceae); black *C. palla* from Pope Valley, Napa Co., CA, where the host plant is *Eurybia radulina* (Asteraceae); red *C. palla* from Twin Bridges, El Dorado Co., CA, where the host plant appears to be *Eurybia integrifolia* (Asteraceae); and *C. hoffmanni* from Truckee, Nevada Co., CA, where the host plant is *Eucephalus breweri* (Asteraceae). Butterflies were frozen within 24 h of capture and remained frozen until 18 h prior to being used in the trial, at which point each individual was rehydrated in a humidity chamber. Freezing is unlikely to have affected the presence of defensive chemicals in these species (Youngken et al. 1951).

### Predators

The palatability and possible mimetic relationship of these three species were tested using European starlings (*Sturnus vulgaris* Linnaeus) as a generalized avian predator. European starlings are known to prey on invertebrates when available, and thus butterflies represent a realistic prey item (Tinbergen 1981; Feare 1984). They have also been used successfully as predators in previous mimicry palatability studies and their visual and learning abilities are well-understood (Van Zandt Brower 1960; Wiklund and Jarvi 1982; Bateson et al. 1995; Ghim and Hodos 2006; Hart and Hunt 2007). The experiment described here was carried out under UC Davis IACUC protocol 16737.

Adult birds were trapped during breeding season in the vicinity of Davis, California, and held in individual cages in an outdoor enclosed aviary where they were exposed to ambient light and temperature. Half of the birds utilized in the study were trapped several weeks before the initiation of the trials, while the other half were trapped approximately 1 year earlier and held communally in the interim. In an effort to ensure that the individuals used in the study are naïve, that is have no prior exposure to the butterfly species under study, they were trapped in an area outside of the butterflies’ ranges. While it is possible that the birds encountered these butterfly species at other times, for example at the natal site or during migration, this is unlikely due to the starlings’ breeding and migration behavior and the phenology of the butterfly flight seasons (Kessel 1957).

### Palatability

We first investigated whether each species of butterfly was palatable to starlings. For the duration of the trial, food was removed from the starlings’ cages at ∼1930 h in order to ensure that the birds had an appetite during the trial. Trials commenced at ∼0630 the following day. Dividers were placed between individual cages in order to prevent the birds from observing each others’ reactions and thus adjusting their own behavior accordingly. Each bird was offered one *P. rapae* (palatable control) and given 5 min to make a decision whether or not to eat the butterfly, at the end of which any remaining pieces of the butterfly were removed from the cage and the day's trial was concluded. Following a 20-min observation period, food was returned to the birds’ cages, where they were allowed to feed ad libitum *,* and cage dividers were removed. This process was repeated for five consecutive days. Any individual that did not eat the palatable control for five consecutive days was excluded from the study.

The remaining twenty birds were randomly assigned to one of the five treatment groups as follows: black *E. chalcedona*, black *C. palla*, red *E. chalcedona*, red *C. palla*, and *C. hoffmanni*. Each treatment group was comprised of four birds. The birds were offered the treatment butterfly species in the same manner as the palatable control for five consecutive days. Because the relevant phenotypic variation occurs almost entirely on the dorsal wing surface, and because all of the test species often bask with the dorsal surface visible, butterflies were presented to the birds with the wings in an open position and with the dorsal surface exposed. We recorded whether the bird ate the entire butterfly, ate the wings but left the body, pecked or handled but did not eat the butterfly, or ignored the butterfly. A prey item may be unpalatable because it is distasteful, toxic, or both (Skelhorn and Rowe 2010). Any response other than ingestion of the entire prey item was considered evidence that the prey item is distasteful to the predator, while responses consisting of predominantly the last two categories were considered to be evidence of the prey item's toxicity. We also recorded overall handling time and/or time until ingestion, as well as any adverse behaviors or reactions by the starlings (vomiting, bill wiping, etc.). Ingestion with long handling time was considered to be evidence of distastefulness, as was excessive bill wiping following ingestion, while vomiting following ingestion was considered to be evidence of toxicity (Van Zandt Brower 1958a; Coppinger 1970; Prudic et al. 2002; Skelhorn and Rowe 2010).

### Mimicry

Once the palatability status of each morph was established, we investigated whether or not there was evidence of a mimetic relationship between any of the species. Because the black form of *E. chalcedona* was the only entity that appeared to be unpalatable (distasteful but not toxic, see Results below), we tested the mimetic relationship between it and the (female) black form of *C. palla*. Upon completion of the palatability phase of the study, the birds from the black *E. chalcedona* treatment group were then given black *C. palla* in the same manner described above for five consecutive days, with the same response variables recorded (Van Zandt Brower 1958a; Prudic et al. 2002; Lindstrom et al. 2006; Skelhorn and Rowe 2010; Hotová Svádová et al. 2013).

### Statistical analyses

We performed all statistical analyses using R (R Development Core Team 2012). First, we assessed the palatability of each butterfly species/morph using a repeated measures mixed model ANOVA, where butterfly morph and trial day number were treated as fixed effects and bird ID was treated as a random effect. For each trial, predator response was scored as an ordinal response variable as follows: 1 = rejection of the prey item; 2 = handled but rejected; 3 = ate wings but rejected thorax/abdomen; 4 = ate entire prey item in >20 sec; 5 = ate entire prey item in <20 sec.

We assessed whether black *C. palla* is an effective mimic of black *E. chalcedona* by comparing whether birds that were preconditioned to reject black *E. chalcedona* were more likely to reject black *C. palla* than birds that were not preconditioned, by performing a repeated measures mixed model ANOVA as described for the assessment of palatability.

## Results

### Palatability

**Figure 2:**
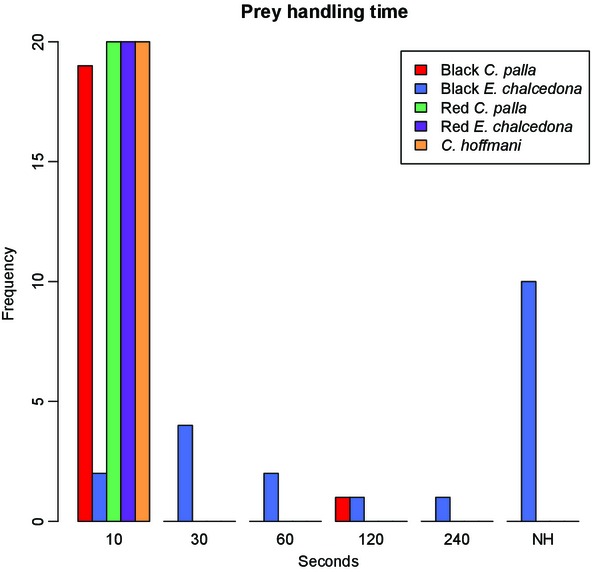
Predator handling time (in seconds) for each butterfly treatment group. Handling times are grouped as follows: 1–10 (10) sec, 11–30 (30) sec, 31–60 (60) sec, 61–120 (120) sec, 121–240 (240) sec, or not handled (NH).

Both color forms of *C. palla*, the red form of *E. chalcedona*, and *C. hoffmanni* were eaten in each instance by all of the birds in the respective treatment groups. None of the birds displayed signs of an adverse reaction or rejection behavior at any point in the study. In each case, the predator consumed the butterfly within 10 sec after it was offered (with one exception: on day 1 of the feeding trials one bird in the black *C. palla* group did not ingest the butterfly until 2 min after it was offered) (Fig.2). We interpret this as an indication that *C. palla* and *C. hoffmanni* are palatable to generalized avian predators, as is the red form of *E. chalcedona*. Therefore, we did not carry out mimicry trials utilizing any of these treatment groups in the role of the model, nor did we investigate the possibility of a mimetic relationship between any of the red-colored entities.

Results from the ANOVA testing palatability are shown in Table2. There was a significant difference in the birds’ response to black *E. chalcedona* versus all other treatment groups (*P*  ≪ 0.01). Test results indicate that this effect changed over time: three of the four birds learned to reject black *E. chalcedona*, while the fourth rejected it on day 3 (ate the wings but rejected the body) but ate it on prior and subsequent days (*P*  = 0.005). While this bird did ingest the butterfly on 4 of 5 days, it also displayed signs of rejection behavior (most notably increased handling time prior to eating and intense bill wiping after consumption) when offered the butterfly each day. Overall, in instances where the predator did handle the prey item, they demonstrated increased handling time compared to the palatable control and the other four treatment groups. We interpret this as evidence that the black form of *E. chalcedona* is distasteful to avian predators, although the penalty does not seem to be a completely effective deterrent to repeated predation in all individuals.

**Table 2:** Results of repeated measures ANOVA of palatability trials testing the effect of type of prey item (*Butterfly*, Fixed Effect), Day (Fixed Effect), and individual predator (*Bird*, Random Effect)

Effects	SS	df	MS	*F*	*P*
(Bird)					
Butterfly***	69.76	4	17.44	14.26	≪0.001
Error	18.35	15	1.223		
(Bird × Day)					
Day***	2.65	1	2.65	11.1	0.005
Butterfly × Day***	13.08	4	3.27	13.72	≪0.001
Error	3.58	15	0.24		
Error	5.1	60	0.09		

*** Indicates *P*  = /<0.005.

None of the birds in the trial vomited or exhibited other signs of ill health at any time. In all cases, the birds ate crickets, mealworms, and regular food immediately after it was offered at the conclusion of the trial. Taken together, the results indicate that black *E. chalcedona* are distasteful but not toxic to a generalized avian predator; this form is unpalatable to the majority of, but not all, individuals, while ingestion in small amounts does not cause emesis or visible negative health effects.

### Mimicry between *E. chalcedona* and *C. palla*

We next conducted trials to see if black *C. palla* is a mimic of black *E. chalcedona*. The results of the ANOVA (Table3) indicate that birds that were preconditioned to reject black *E. chalcedona* were more likely to reject *C. palla* than birds that were not preconditioned to reject *E. chalcedona* (*P*  = 0.03). We considered this to be evidence that black *C. palla* is an FPM mimic of black *E. chalcedona*. However, birds that had rejected black *E. chalcedona* were sometimes willing to attack black *C. palla*, suggesting that they may be able to at least partially discriminate between the two in this setting.

**Table 3:** Results of repeated measures ANOVA of mimicry trials testing whether predators preconditioned to reject black forms of the model rejected black forms of the mimic more often than unconditioned predators

Effects	SS	df	MS	*F*	*P*
(Bird)					
Butterfly*	60.02	1	60.02	8.121	0.03
Error	44.35	6	7.39		
(Bird × Day)					
Day	1.8	1	1.8	1.5	0.27
Butterfly × Day	3.2	1	3.2	2.67	0.15
Error	7.2	6	1.2		
Error	7.4	24	0.31		

* Indicates *P*  < 0.05.

## Discussion

Results of our palatability trials clearly indicate that all butterfly entities tested are palatable to a generalized avian predator, with the exception of the black form of *Euphydryas chalcedona*. To our knowledge, the palatability of *Chlosyne hoffmanni* has not previously been tested and these results suggest that this species does not play the role of the model in any mimicry systems in this study area, nor does it act as a mimic in this system. The results from the *Chlosyne palla* tests confirm those from field trials conducted by Ley and Watt (1989), although it should be noted that the Colorado population tested in the latter study is monomorphic (red-orange background) and utilizes a different host plant (*Erigeron speciosus macranthus*, Asteraceae) than the populations in our study. There have been no prior trials addressing whether the polymorphic populations and in particular the black females of *C. palla* are palatable, an important step in the characterization of this mimicry system.

The palatability results from the *E. chalcedona* trials shed new light on a previously conducted study (Bowers 1981). While we found that black *E. chalcedona* utilizing *Scrophularia californica* as a larval host plant are indeed unpalatable (as did Bowers), our results indicate that the red-orange form, which utilizes *Penstemon newberryi* as a larval host plant, is palatable to a generalized avian predator. Bowers’ (1981) study tested larvae raised on *S. californica* and on *Keckiella antirrhinoides*, but did not tested larvae raised on *Penstemon newberryi*.

Unpalatability in Lepidoptera is often derived from chemicals sequestered from their larval host plants (Rothschild 1972; Blum 1981; Brower 1984; Bowers 1988). While no such chemicals have been described for these *Chlosyne* host plant species, this absence of evidence could not be taken as proof that these butterfly species are indeed palatable, as cases of autogenous chemical defenses in Lepidoptera are not uncommon (Bowers 1992). However, taken together with the results of the feeding trials, it seems unlikely that either of these species manufacture defensive chemicals de novo or sequester them from their respective larval host plants.

In the case of the *E. chalcedona* host plants, however, the results are less straightforward. The host plant species utilized by the black populations (*S. californica*) has been shown to contain iridoid glycosides in the form of aucubin, (Kooiman 1970) while the other host plant used by Bowers (1981), *K. antirrhinoides*, contains catalpol, as does *P. newberryi*, used by the red populations in this study (Kooiman 1970). A study by Bowers (1986) showed that when an *E. chalcedona* population that normally utilizes an aucubin-producing host plant (*K. breviflora*) and one that utilizes a catalpol-producing host plant (*P. newberryi*) are fed the “wrong” plant, larvae from the latter population performed significantly worse (in terms of growth and survival) than when fed on their usual host plant. Further studies are necessary to determine whether this is related to these populations’ respective abilities to sequester these different types of iridoid glycosides as larvae and whether this in turn is responsible for the difference in palatability.

Although our results fail to provide support for the coevolutionary chase hypothesis (Huheey 1988; Joron and Mallet 1998), they do suggest an alternative explanation for the observed phenotypic polymorphism. While the black form of *E. chalcedona* clearly seems to display an honest aposematic signal advertising distastefulness, the red form appears to be either a dishonest aposematic signal (possibly indicating its role as a mimic in an as yet undescribed system) or does not in fact represent aposematism at all. We are not aware of any species that are sympatric with the red form of *E. chalcedona* that would be potential models for this phenotype. While several hypotheses have been put forth to explain variation in aposematic phenotype, as far as we are aware, this is the first evidence of variation in model phenotype correlated with variation in palatability (Stevens and Ruxton 2012).

Our results support the proposed hypothesis of female-limited polymorphic mimicry between the black forms of the *E. chalcedona* and *C. palla* (Shapiro and Manolis 2007). While experienced predators were sometimes willing to attack and ingest the mimic, they rejected *C. palla* significantly more often than predators with no preconditioning. Some birds appeared to be able to sometimes discriminate between the two species; however, the convergent phenotype of black female *C. palla* appears to sufficiently resemble the model such that experienced predators reject both species (based on visual cues) more often than they consumed them. Avian and insect vision are different from human vision, so it seems likely that both the predators and prey interpret the butterflies’ visual signals differently than humans do (Swihart 1970; Feare 1984; Arikawa et al. 1987; Hart et al. 1998; Ghim and Hodos 2006; Stavenga and Arikawa 2006; Hart and Hunt 2007; Briscoe 2008). The vision of *S. vulgaris* has been shown to be typical of most bird species studied, and thus their response to this visual signal is likely to be similar to that of other avian predators that these butterflies might encounter (Hart et al. 1998).

We found no evidence that European starlings harbor innate aversion to any of the butterflies involved, including the aposematic, unpalatable model. In each of the treatment groups, the predator was initially willing to attack the butterfly in under 10 sec. Only after repeated exposure did the birds begin to reject or hesitate in attacking black *E. chalcedona*. Therefore, some other selective mechanism involving visual signaling must contribute to the evolution of this butterfly phenotype. The aposematic warning coloration exhibited by black *E. chalcedona* shares similar components with other species that exhibit aposematism (*Adelpha californica* and *Euphydryas phaeton* in butterflies, the white and black striping of skunks, etc.) and is consistent with observations that high color contrast and/or high luminance contrast often functions as an effective aposematic signal (Ruxton et al. 2004; Prudic et al. 2006).

While the predators’ reactions to the palatable butterflies were remarkably consistent (attack and ingestion in under 10 sec in all but one trial), we did observe individual variation in response to the unpalatable black *E. chalcedona*. This type of individual variation is common in palatability trials (Van Zandt Brower 1958a,c; Ritland 1991; Prudic et al. 2002), although it seems to be diminished in cases where the prey is extremely unpalatable (Van Zandt Brower 1958b). While *E. chalcedona* does not appear to be as noxious as some models, for example *Battus philenor*, it does appear to be consistently distasteful to a generalized avian predator (Van Zandt Brower 1958b; Fordyce 2000; Sime et al. 2000). Despite the fact that one bird in the black *E. chalcedona* treatment group ingested the prey in four of five trials, all of the birds in that treatment group showed increased handling time after the first trial as well as adverse reactions (excessive bill wiping, a previously cited negative reaction in response to distasteful prey)(Van Zandt Brower 1958a) after all trials. Furthermore, in almost all of these trials, the birds’ initial contact with the butterfly was by pecking at the wing, and this went on for several seconds before the predator eventually ate or rejected the prey. This suggests that had the trial been conducted using live butterflies (or had it occurred in the wild), the butterflies may have had an opportunity to escape. This has been previously discussed by Wiklund and Jarvi (1982) and Ritland (1991), suggesting that individual-level selection may thus play a greater selective role in maintaining mimicry systems than is usually considered.

There are alternative explanations that may explain the observed phenotypic patterns; however, none are convincing. Selection based on thermoregulatory coloration would produce the opposite elevational distribution than what is observed here, and this hypothesis does not explain the female-limited polymorphism in *C. palla* (Watt 1968; Kingsolver 1983, 1988; Boggs and Murphy 1997). Evidence suggests that pigmentation may provide protection against strong sunlight at high elevation; however, this poses the same problem as those described for thermoregulation (Nijhout 1991; Halder and Bridgeman-Shah 1995; Gloster and Neal 2006; Brenner and Hearing 2008; Cooper 2012).

The level of individual variation that we observed in the predators’ responses indicates that further investigation with additional predators (numerically as well as with additional species) is required to accurately assess the level of protection that both the model and the mimic receive in this system. We therefore urge caution in interpretation of these results, as the small sample size may mask or inflate the level of unpalatability of black *E. chalcedona*, as well as the mimetic resemblance of black *C. palla*. However, the entirety of the evidence (the results presented here, as well as the results of previous studies including the quantitation of host plant phytochemicals) suggests that the difference between the true variation and the observed variation is likely to be a one of magnitude rather than direction.
